# Supplementary material for: Polyglutamine-Expanded Androgen Receptor Alteration of Skeletal Muscle Homeostasis and Myonuclear Aggregation Are Affected by Sex, Age and Muscle Metabolism
Source: Cells. 2020 Jan 30;9(2):325. doi: 10.3390/cells9020325 (PMC7072234; doi:10.3390/cells9020325)
Supplement: Supplementary file 1 [file cells-09-00325-s001.pdf]

Table S1. Primers Real Time used for quantitative PCR analyses.

Mouse	Forward (5'-3')	Reverse (5'-3')
B-Actin	GACAGGATGCAGAAGGAGATTACTG	CTCAGGAGGAGCAATGATCTTGAT
Foxo3a	CGCTGTGTCCCTACTTCA	CCCGTGCCTTCATTCTGA
LC3b	CACTGCTCTGTCTTGTGTAGGTTG	TCGTTGTGCCTTTATTAGTGCATC
Musa1	TCGTGGAATGGTAATCTTGC	CCTCCCGTTTCTCTATCACG
Smart	TCAATAACCTCAAGGCGTTC	GTTTTGCACACAAGCTCCA
Myog	CTTGCTCAGCTCCCTCAAC	TGGGAGTTGCATTCACTGG
Myh3	GGGACCTTGCCAAGAAGAA	GTCGTTCCTCACGGTCTTG
Musk	ATCACCACGCCTCTTGAAAC	TGTCTTCCACGCTCAGAATC
Myh8	GAGGGCATCCGCATCTG	GATGAACTGTCCCTCTGGAATAG
Achrg	AGTGCAGGCAGTATTGGAGA	AGGTTACAGGCATCCACACAG
Ncam	ACAATGCTGCGAACTAAGGA	TGCCACTTGACACAGGA
Opa1	ATACTGGGATCTGCTGCTGTTGG	AAGTCAGGCACAATCCACTT
Dnm1l	TCAGATCGTCGTAGTGGGAA	TCTTCTGGTGAAACGTGGAC
Pparg1a	ATAAGTCCTTCCCGCTGAC	GGAATTCATGTCGTAGATGACAAAT
Sqstm	CCCAGTGTCTTGGCATTCTT	AGGGAAAGCAGAGGAAGCTC
Clcn1	GTCCTCAGCAAGTTTAT GTCC	GAATCCTCGCCAGTAAT TCC

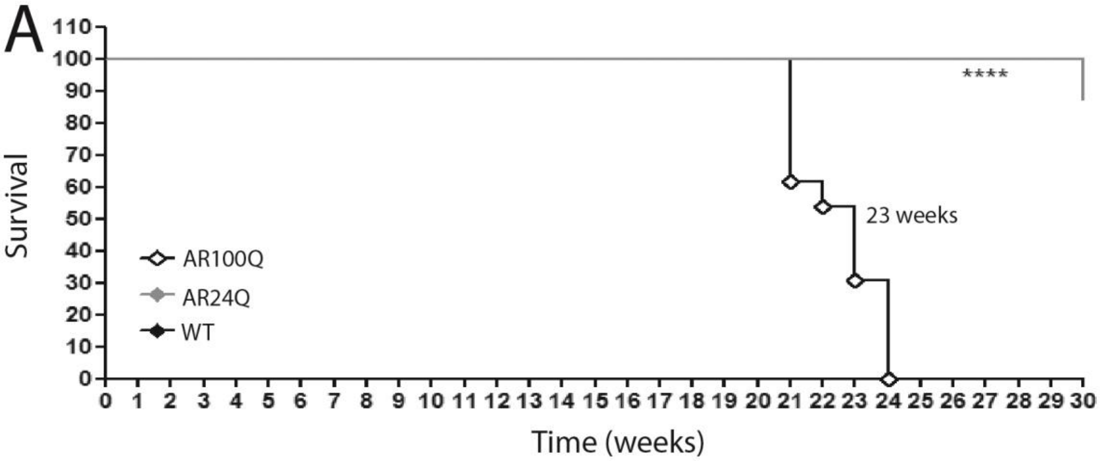

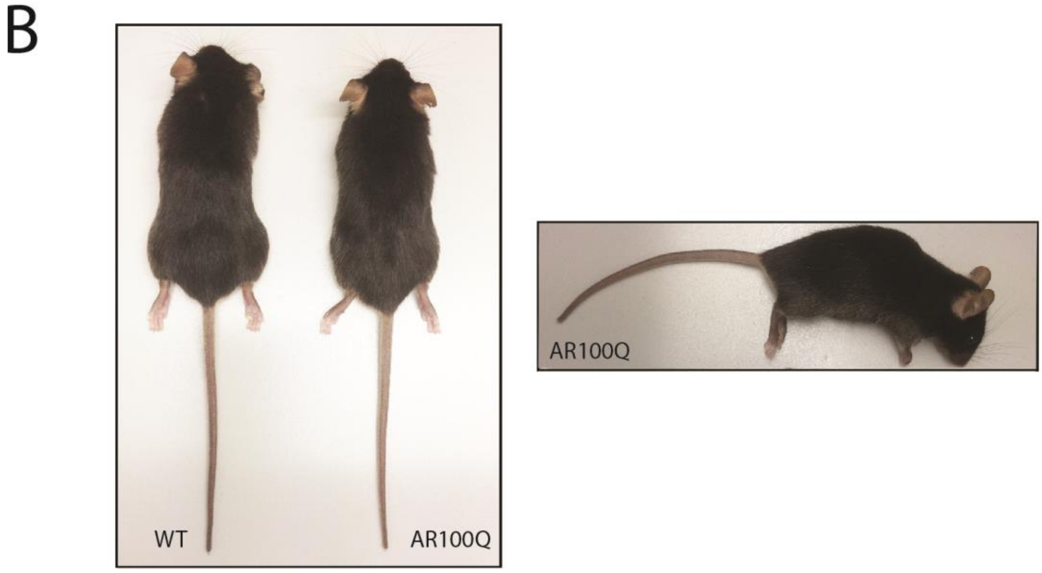

Figure S1. Decreased lifespan of female AR100Q mice and pictures of male mice. (**A**) Kaplan-Meier analysis of lifespan of WT (*n* = 13), AR24Q (*n* = 8), and AR100Q (*n* = 13) female mice. Survival analysis revealed that the overexpression of non-expanded AR does not affect lifespan in females, whereas overexpression of polyQ-expanded AR causes premature death with a median survival of 23 weeks. Survival curves were compared using Log-rank (Mantel-Cox) test. (**B**) Representative pictures of 8-week-old WT and AR100Q male mice.

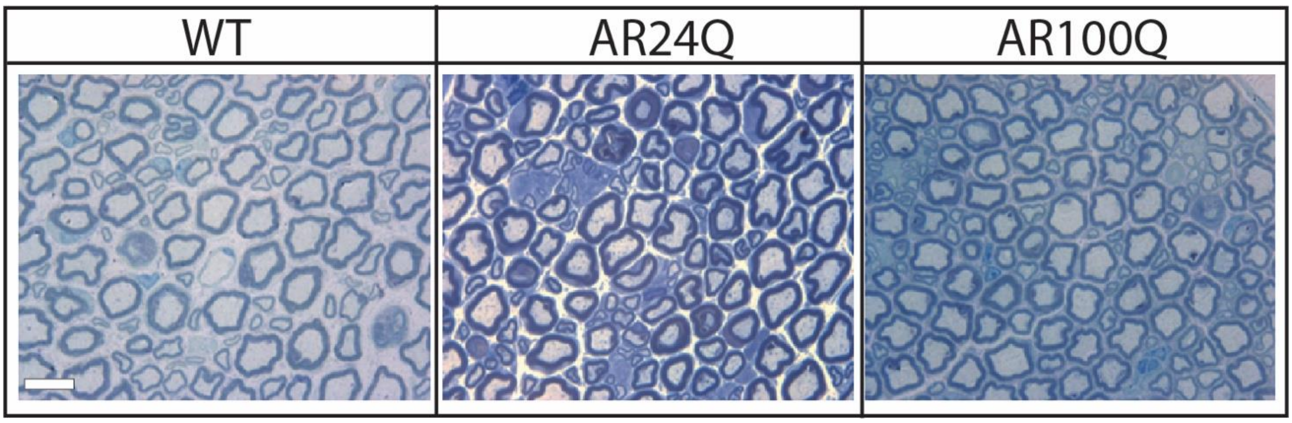

Figure S2. Absence of peripheral nerve pathology in AR24Q and AR100Q mice. Toluidine blue staining of semi-thin femoral nerve transversal sections of 8-week-old WT, AR24Q, and AR100Q male mice (*n* = 3). Bar, 10 micron.

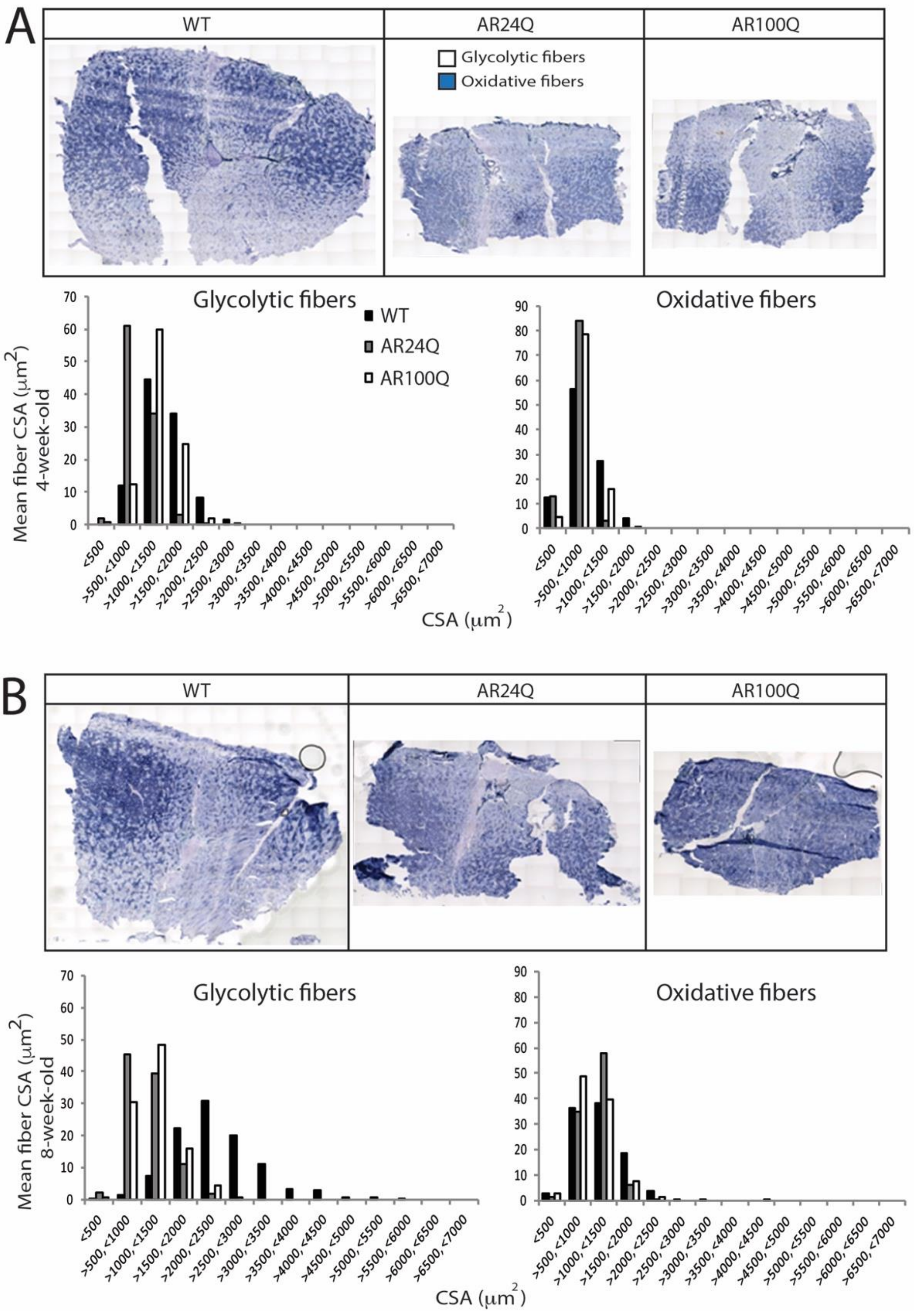

Figure S3. Atrophy of glycolytic fibers exceeds that of oxidative fibers in AR100Q mice. (**A**-**B**) Analysis of the glycolytic and oxidative fiber distribution in the gastrocnemius muscle of (**A**) 4-week-old and (**B**) 8-week-old WT, AR24Q, and AR100Q male mice (n = 3). Number of fibers (4-week-old): 1224 WT, 1224 AR24Q, and 1237 AR100Q. Number of fibers (8-week-old): 1232 WT, 1257 AR24Q, and 1219 AR100Q.

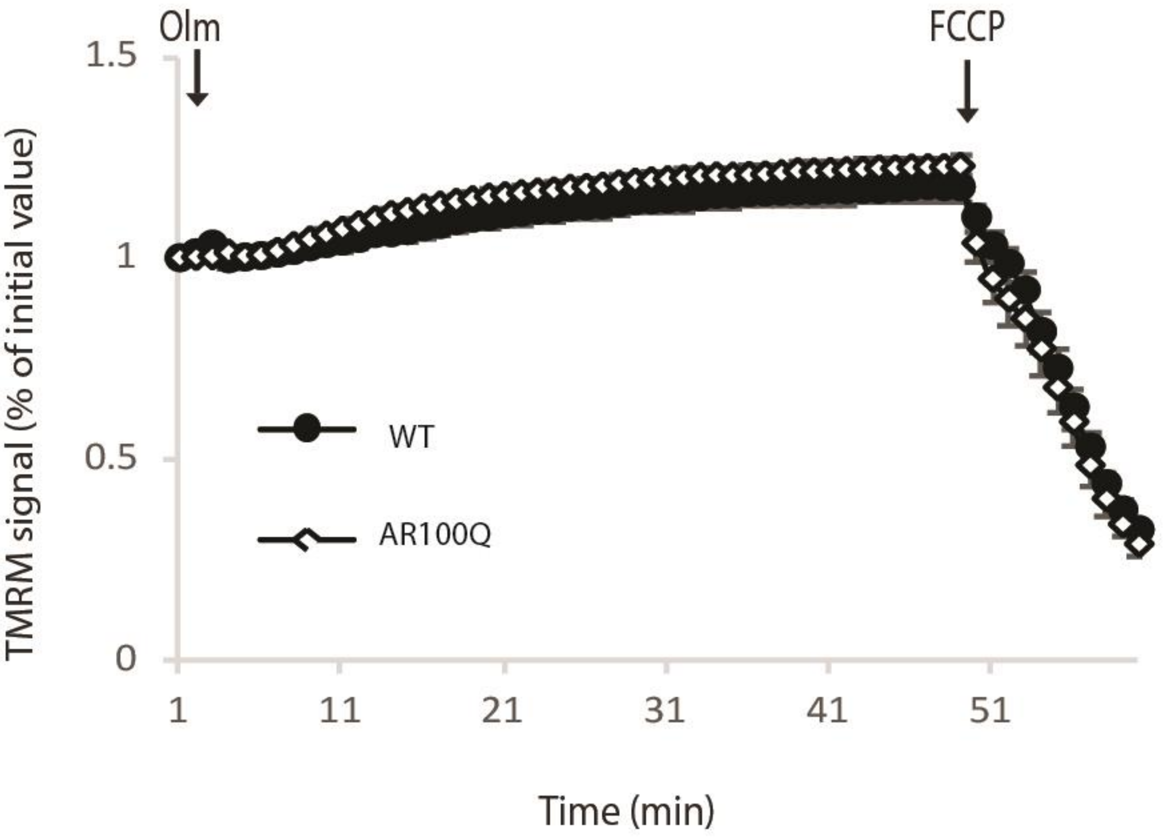

Figure S4. Mitochondrial depolarization is age-dependent in AR100Q mice. Mitochondrial membrane depolarization measured in fibers isolated from flexor digitorum brevis (FDB) of 4-week-old WT and AR100Q male mice (n = 3). Olm, oligomycin; FCCP, protonophore carbonyl cyanide *p*-trifluoromethoxyphenylhydrazone TMRM, tetramethyl rhodamine methyl ester.

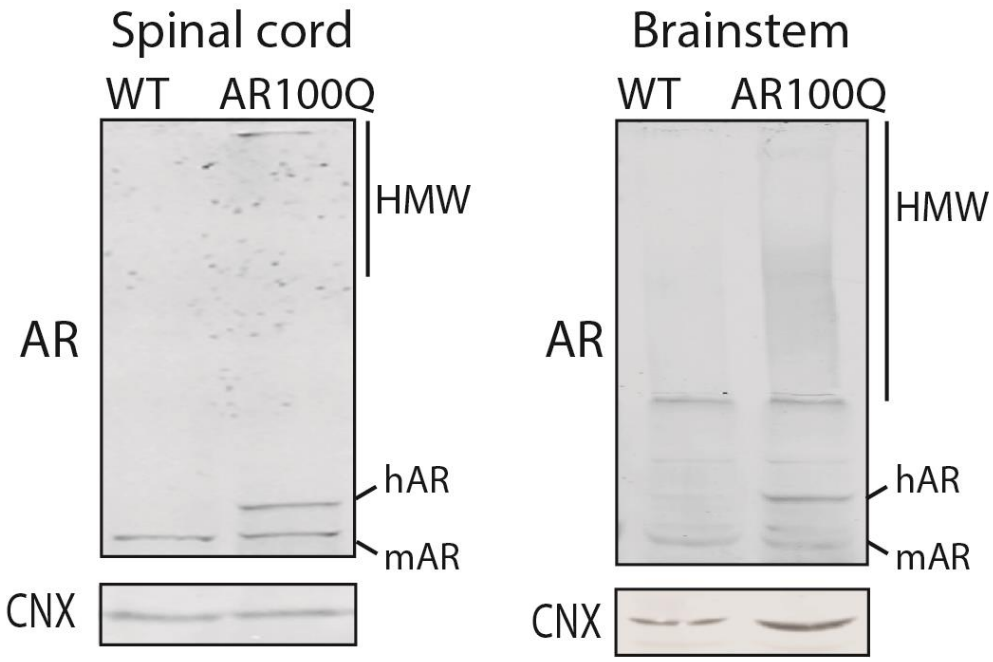

Figure S5. PolyQ-expanded AR forms aggregates in spinal cord and brainstem. Western blotting analysis of AR aggregation in the indicated tissues of 8-week-old AR100Q male mice. Total protein lysates were obtained using a RIPA buffer containing 0.1% SDS. Aggregation was increased by 1.1- and 1.3-fold in the spinal cord and brainstem of AR100Q mice compared to WT mice. AR was detected with a specific antibody, and calnexin (CNX) was used as loading control (*n* = 3 mice). HMW, high-molecular weight species. Shown is one experiment representative of three.

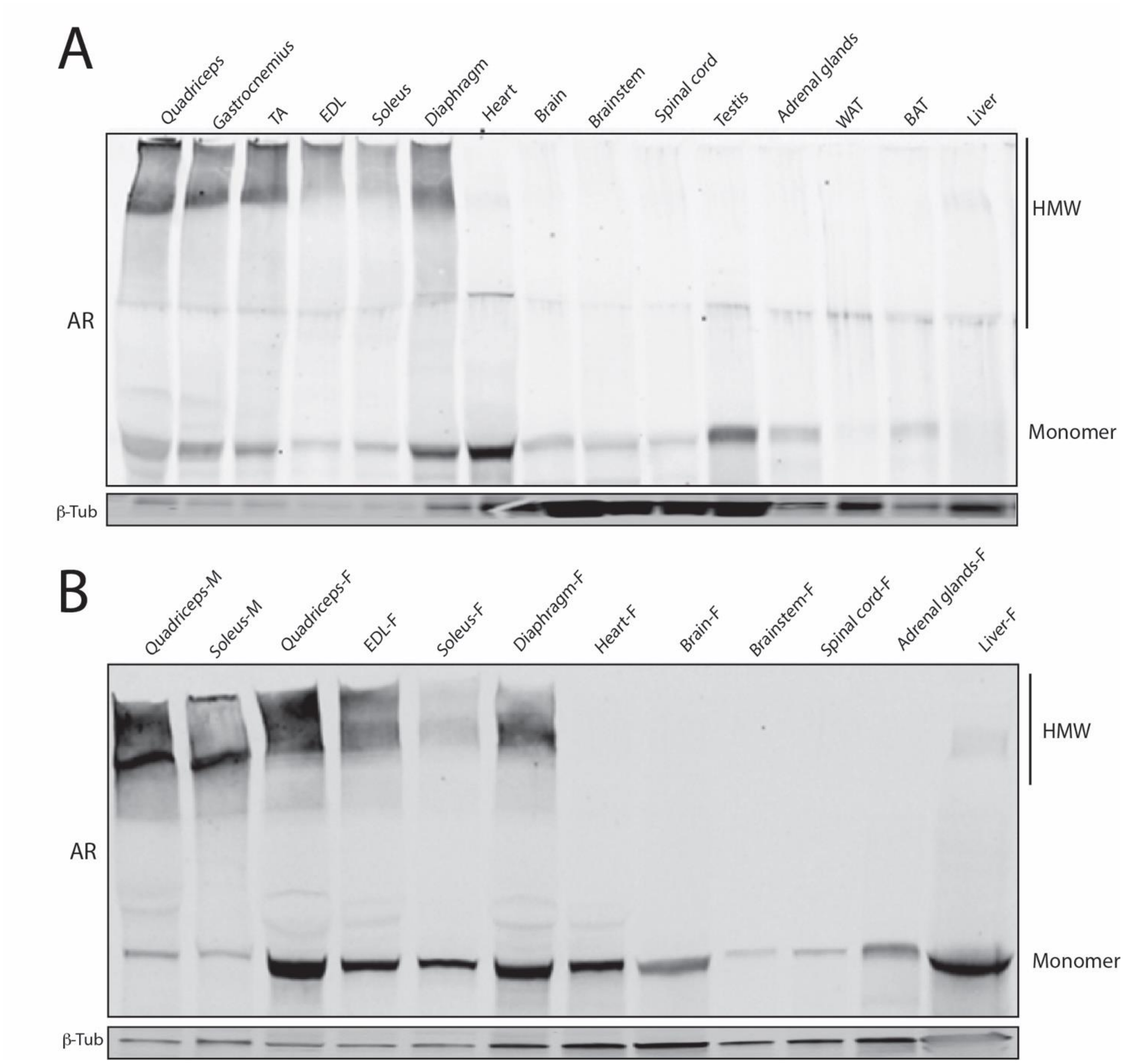

Figure S6. PolyQ-expanded AR forms 2% SDS-resistant aggregates selectively in skeletal muscle. (**A**) Western blotting analysis of AR aggregation in 8-week-old AR100Q male mice. (**B**) Western blotting analysis of AR aggregation in 8-week-old male (M) and female (F) AR100Q mice. Total protein lysates were obtained using a RIPA buffer containing 2% SDS. AR was detected with a specific antibody, and beta-tubulin (β-Tub) was used as loading control (n = 3 mice). HMW, high-molecular weight species. Shown is one experiment representative of three.

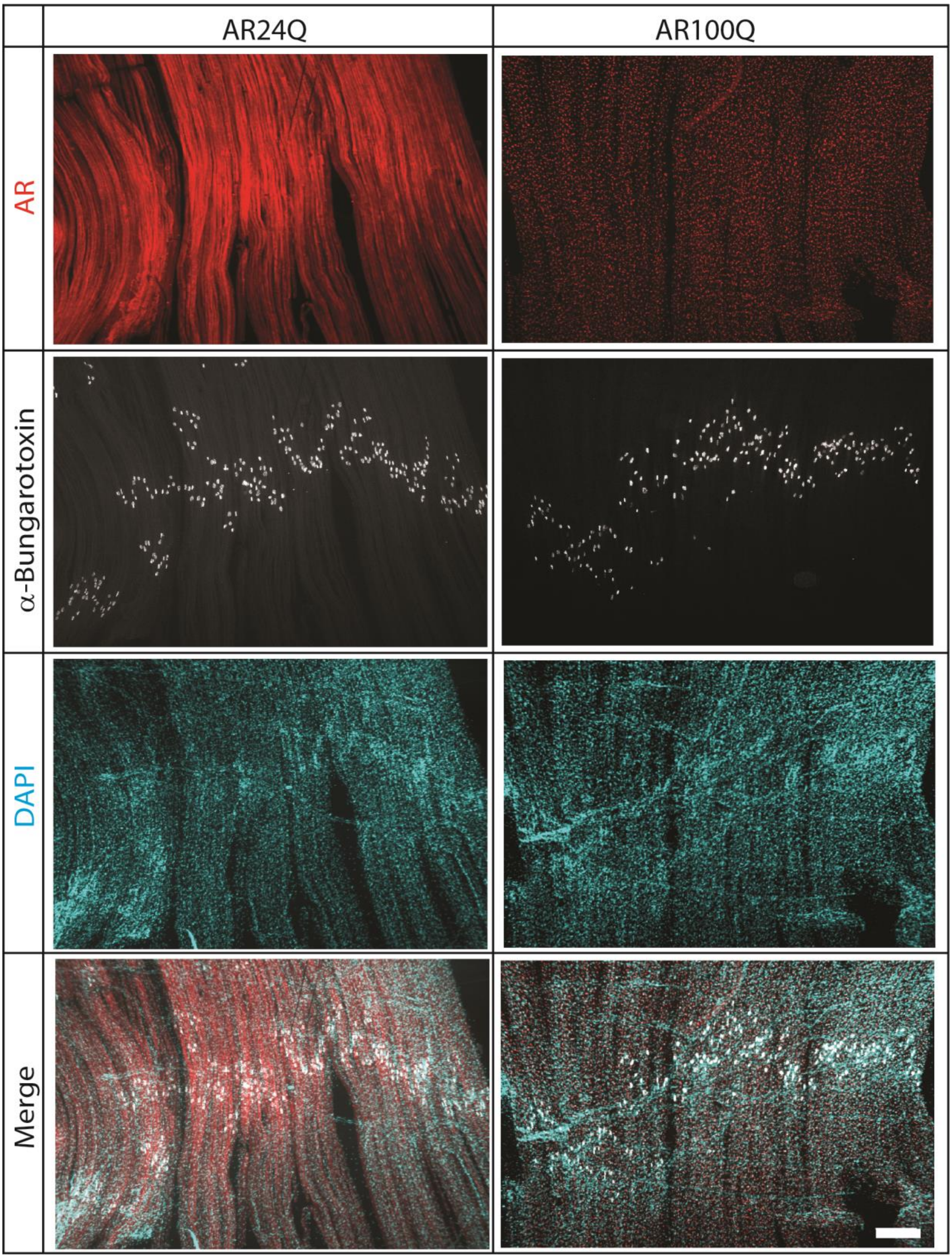

Figure S7. PolyQ-expanded AR forms inclusion bodies in the muscle of AR100Q mice. Fluorescent microscopy analysis of AR subcellular localization in intact myofibers from the levator auris longus (LAL) muscle of 8-week-old AR24Q and AR100Q male mice. Bar, 250 micron. AR was detected with a specific antibody (red), NMJs by staining with α-Bungarotoxin (white), and nuclei with DAPI (blue). Shown are representative images of at least 3 mice.

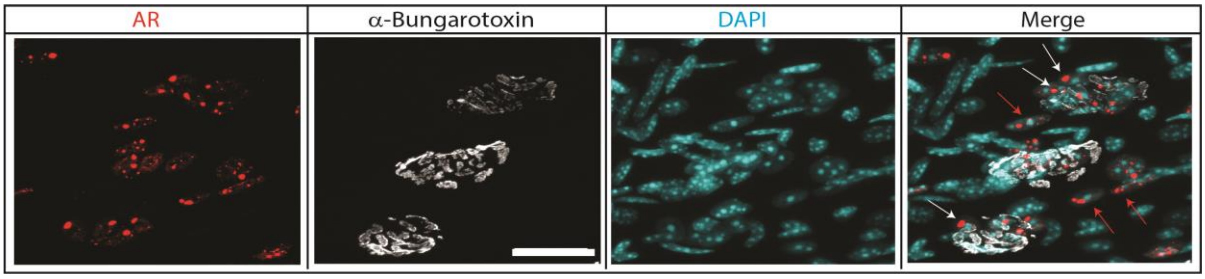

Figure S8. PolyQ-expanded AR-positive inclusion bodies detected in sub-synaptic and non-sub-synaptic nuclei. Immunofluorescence analysis of AR (red), NMJs (white), and DAPI (blue) in intact fibers from LAL muscle of 8-week-old AR100Q male mice. Red arrows: non-synaptic myonuclei, white arrows: synaptic myonuclei. Bar, 25 micron. AR was detected with a specific antibody (red), NMJs by staining with α-Bungarotoxin (white), and nuclei with DAPI. Shown are representative images of at least 3 mice.

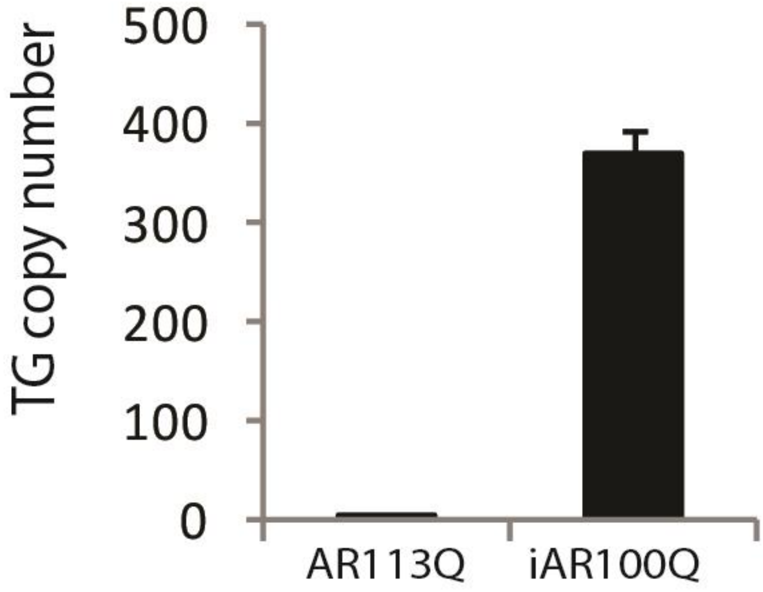

Figure S9. Copy number of *hAR* transgene in iAR100Q mice. Analysis of gene copy number by quantitative PCR. AR113Q knock-in mice, in which the endogenous *AR* exon 1 was replaced with the human exon 1, were used as reference and normalized to 1. Graph, mean ± sem, $n = 3$.

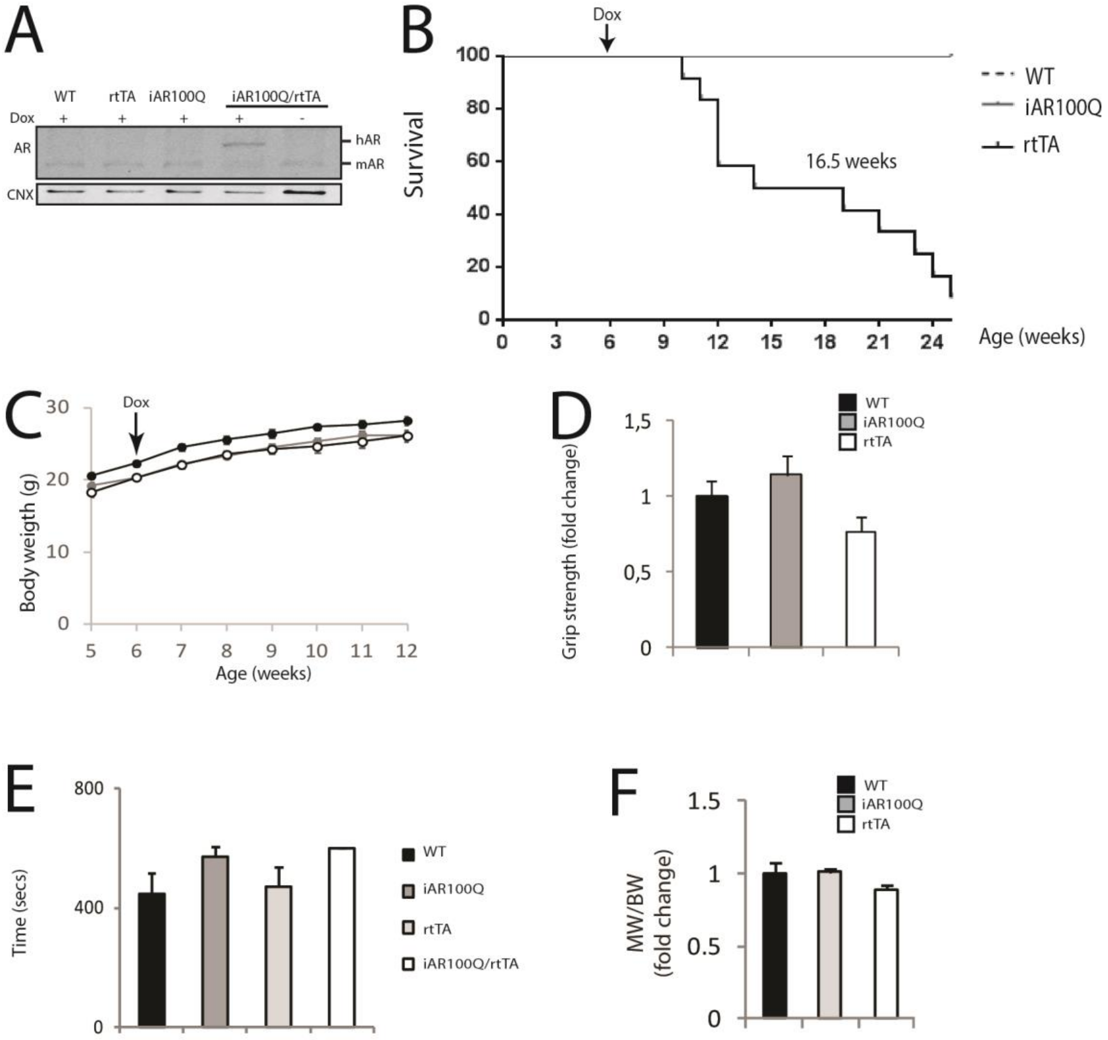

Figure S10. Generation and characterization of inducible transgenic mice expressing AR100Q. (**A**) Western blotting analysis of AR levels in 8-week-old WT (*n*= 3), rtTA (*n*= 3), iAR100Q (*n*= 3), iAR100Q/rtTA (*n*=3) male mice treated with either vehicle (sucrose 50g/l) or doxycycline (sucrose 50g/l, dox=1g/l). (**B**) Kaplan-Meier survival curves of WT (*n* = 8), rtTA (*n* =12), and iAR100Q (*n* = 8) male mice treated with dox. Survival curves were compared using Log-rank (Mantel-Cox) test. Although dox treatment reduced the median survival of the rtTA mice to 16.5 weeks, the effect of induction of expression of AR100Q on survival was statistically different from that of rtTA mice. (**C**) Temporal changes in mean BW of WT (*n* = 8), rtTA (*n* = 8), and iAR100Q (*n* = 8) male mice treated with dox. (**D**) Grip strength analysis of muscle force of WT (*n* = 8), rtTA (*n* =8), and iAR100Q (*n* = 8) mice treated with dox. (**E**) Rotarod task performance in male mice (*n* = 3-8). (**F**) Quadriceps MW normalized to BW of WT (*n* = 5), rtTA (*n* = 5), and iAR100Q (*n* =5) mice treated with dox. Graph, mean ± sem, (**D** and **E**) one-way ANOVA followed by Tukey's Honest Significant difference post hoc test.

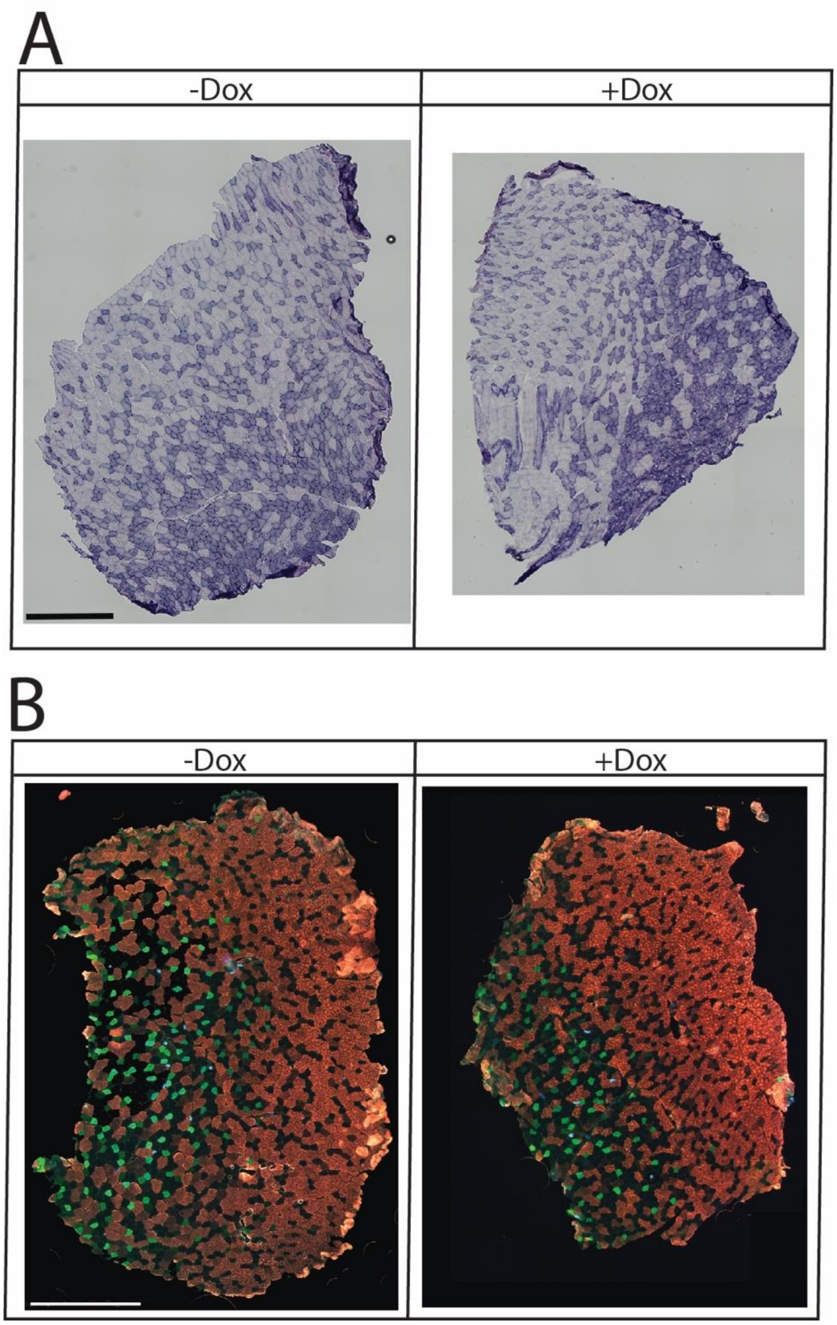

Figure S11. Two-week expression of polyQ-expanded AR in the adulthood does not cause metabolic alterations and fiber-type changes in muscle. (**A**) NADH analysis of TA of 8-week-old iAR100Q male mice (*n* = 3) treated with vehicle (-Dox) and Dox (+ Dox). Shown are representative images. Bar, 500 micron. (**B**) Immunofluorescence analysis of MyHC type I (blue), IIa (green), IIx (black), and IIb (red) from 8-week-old iAR100Q male mice (*n* = 3) treated with vehicle (-Dox) and Dox (+ Dox). Shown are representative images. Bar, 500 micron.
